# Inflammation increases the penetrance of behavioral impairment in *Shank3* haploinsufficiency mice – can it explain the behavioral regression in Autism?

**DOI:** 10.1101/2025.08.07.669241

**Published:** 2025-08-11

**Authors:** Sheng-Nan Qiao, Sung Eun Wang, Kun-Yong Kim, Sungsin Jo, Yong-Hui Jiang

**Affiliations:** 1 Department of Genetics, Yale University School of Medicine, New Haven, CT, USA; 2 Department of Neuroscience, Yale University School of Medicine, New Haven, CT, USA; 3 Department of Pediatrics, Yale University School of Medicine, New Haven, CT, USA; 4 Department of Obstetrics, Gynecology & Reproductive Sciences, Yale University School of Medicine, New Haven, CT, USA; 5 Department of Biology, Soonchunhyang University, Soonchunhyang-ro 22, Sinchangmyeon, Asan, South Korea

## Abstract

Behavioral regression occurs in ~40% of *SHANK3*-associated autism spectrum disorder (ASD). We previously reported that significant behavioral regression in a small cohort with *SHANK3* haploinsufficiency patients, triggered by subclinical infections, responded to immunomodulator treatments. We hypothesize that behavioral regression results from the interplay between *SHANK3* deficiency and neuroinflammation. Using *Shank3* exon 4–22 deletion heterozygous mutant (*Sh3*^+/−^) mouse, which shows no significant behavior impairments, we established a preclinical model – *Shank3* haploinsufficiency mouse undergoing systemic inflammation challenge via intraperitoneal injection of lipopolysaccharides (LPS). We found that, two weeks after LPS challenge, wild-type mice (WT) recovered but *Sh3*^+/−^ mice exhibited motor impairment, anxiety-like behaviors, and excessive grooming, similar to *Shank3* exon 4–22 deletion homozygous mutants. Anti-inflammatory treatment partially reversed LPS-induced behavioral changes. Transcriptomic analysis revealed upregulation of neuroinflammation-related genes and downregulation of synaptic function-related genes in *Sh3*^+/−^ mice in response to LPS. Especially, pro-inflammatory genes and microglia markers were overly activated that may result from the increased Toll-Like Receptor 4 (TLR4) expression in microglia in *Sh3*^+/−^ mice. Our findings indicate that neuroinflammation increases the penetrance of behavioral impairment in *Shank3* haploinsufficiency mice and support a potential mechanism for the behavioral regression in human *SHANK3* disorders for future investigations.

## Introduction

Autism spectrum disorder (ASD) is a group of neurodevelopmental conditions with typical onset before the age of three ^1^. A significant proportion of children with ASD shows developmental arrest at an early developmental age and behavioral regression throughout the lifespan. Behavioral regression is characterized by loss of previously acquired skills, including loss of purposeful hand use, loss of acquired speech, and gait abnormalities ^2–4^. Catatonic features are frequently associated with behavioral regression observed in ASD ^5,6^. However, the underlying mechanism for the early developmental arrest and behavioral regression remains poorly understood.

The *SHANK3* mutation is one of the most common monogenic defects associated with ASD in humans ^7–10^. Genetic mutations in *SHANK3* gene can manifest as Phelan-McDermid Syndrome (PMS), characterized by a chromosomal deletion of 22q13.3 or single nucleotide variants, indels, exonic deletions within the *SHANK3* gene in individuals with primary ASD ^11^. Patients with *SHANK3* haploinsufficiency also exhibit severe intellectual disability, motor deficits, and speech delays ^12–14^. Developmental regression with catatonic features has been reported in ~40% of cases with *SHANK3* haploinsufficiency ^15–20^. We previously described four patients with pathogenic *SHANK3* mutations who experienced acute and severe behavioral regression following possible subclinical viral infections ^21^. Notably, treatment with intravenous immunoglobulins (IVIG) combined with anti-inflammatory drugs and immunomodulators significantly reversed the behavioral regression in these cases ^21^. This clinical report strongly suggests a potential link between inflammation and behavioral regression associated with *SHANK3* haploinsufficiency. However, the direct causality could not be investigated and determined from human studies.

SHANK3 protein is primarily characterized as a scaffold protein located at the postsynaptic density of neurons, interacting with other synaptic proteins and affecting synaptic function ^22^. However, the role of SHANK3 in non-neuronal cells such as microglia has been poorly studied ^23–26^. Microglia activation is an important part of the innate immune response in the brain and has been reported in ASD patients ^27^. Our previous studies showed that inflammation induces acute behavioral changes ^28^ and aggravates hypothermia in *Shank3* mutant mouse model ^29^. These initial findings suggest a potential mechanistic link between *SHANK3* deficiency and inflammatory response.

Our lab previously generated a *Shank3* mutant mouse line with a deletion of exon 4 to 22 (*Shank3*^*Δe4–22*^) that disrupts most of the *Shank3* coding region^30^. *Shank3*^*Δe4–22*^ homozygous but not heterozygous mice showed robust face validity for autism-like behaviors^30^. Therefore, *Shank3*^Δe4–22^ heterozygous mice offer a unique opportunity to examine the interaction between genes and environment, specifically whether neuroinflammation may modify the penetrance of behavioral phenotypes associated with *SHANK3* deficiency in humans. In this study, we utilized the *Shank3*^Δe4–22^ heterozygous mice and investigated the behavioral outcome, transcriptomic profiles, and microglia activation after systemic inflammatory challenge induced by lipopolysaccharides (LPS). These findings provide a general insight on the role of neuroinflammation in ASD susceptibility.

## Results

### Systemic inflammation via LPS modified the penetrance of motor dysfunction, anxiety-like behavior, and repetitive behavior in *Shank3*^Δe4–22^ heterozygous (*Sh3*^+/−^) mice.

**Figure 1.**
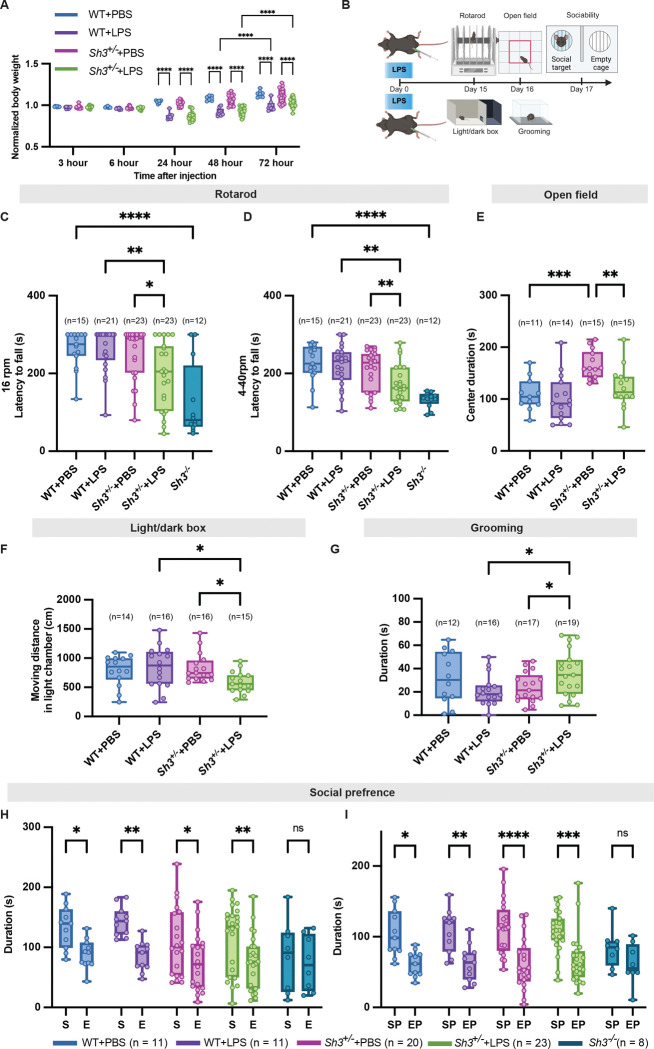
LPS challenge induced selective behavioral impairments in *Sh3*^+/−^ mice. **A. The body weight loss after LPS intraperitoneal injection.** Mouse was weighed before injection, and at 3, 6, 24, 48, and 72 hours after LPS (1 mg/kg) or the same volume of PBS injection. Each mouse’s body weight was normalized to the weight before injection. Two-way repeated measures ANOVA was performed (genotype/LPS effect F _(3, 50)_ = 34.32, *p* < 0.0001; Hour effect F _(4, 200)_ = 84.15, *p* < 0.0001; genotype/LPS X Hour interaction F (12_, 200)_ = 22.64, *p* < 0.0001). Post-hoc Tuckey’s test showed no significant difference between WT+PBS (n = 8) and *Sh3*^+/−^+PBS (n = 17) or between WT+LPS (n = 8) and *Sh3*^+/−^+LPS (n = 21) at 3 hours, 6 hours, 24 hours, 48 hours and 72 hours after injection. Mouse body weight started to reduce significantly after LPS injection compared to PBS injection at 24 hours, 48 hours and 72 hours. Mouse body weight from LPS groups significantly increased at 72 hours compared to 48 hours after injection in both WT+LPS and *Sh3*^+/−^+LPS groups. See multiple comparison statistics in Supplementary Table 5. B. Schematic drawing for the experimental design. **C-D. LPS challenge impaired motor function in *Sh3***^**+/−**^ **mice.** Steady speed rotarod (16 rpm, **C**) and accelerated speed rotarod (4–40 rpm, **D**) tests were performed and latency to fall was measured. No significant difference was found between WT+PBS and *Sh3*^+/^+PBS (16 rpm, *p* =0.5487, 4–40 rpm, *p* =0.3283) or between WT+PBS and WT+LPS (16 rpm, *p* =0.9797, 4–40 rpm, *p* =0.6711). *Sh3*^+/−^+LPS significantly reduced latency to fall compared to *Sh3*^+/−^+PBS (16 rpm, *p* =0.0126, 4–40 rpm, *p* =0.0061) or compared to WT+LPS (16 rpm, *p* =0.002, 4–40 rpm, *p* =0.001). *Sh3*^−/−^ mice significantly reduced latency to fall compared to WT+PBS (16 rpm, *p* < 0.0001, 4–40 rpm, *p* < 0.0001). **E-F. LPS challenge increased anxiety-like behavior in *Sh3***^**+/−**^ **mice.** Mouse duration spent in the center of the open field test and travel distance in light chamber of light/dark box test were measured. No significant difference found between WT+PBS and WT+LPS (OFT, *p* = 0.5956, light/dark box *p* = 0.6004). *Sh3*^+/−^+LPS showed less center duration than *Sh3*^+/−^+PBS (*p* = 0.0033) despite that *Sh3*^+/−^+PBS showed increased center duration than WT+PBS (*p* = 0.0005), and *Sh3*^+/−^+LPS showed less travel distance in light chamber than *Sh3*^+/−^+PBS (*p* = 0.0158) or WT+LPS (*p* = 0.0107). **G. LPS challenge increased repetitive behavior in *Sh3***^**+/−**^ **mice.** Grooming behavior was hand scored and compared. *Sh3*^+/−^+LPS showed longer grooming duration than *Sh3*^+/−^+PBS (*p* = 0.0348) or WT+LPS (*p* = 0.0135). **H-I. LPS challenge had no effect on sociability *Sh3***^**+/−**^ **mice.** Three-chamber tests were conducted and duration spent in mouse chamber (S), empty chamber (E), proximity regions in mouse chamber (SP) and empty chamber (EP) were compared (Two-way repeated measures ANOVA. **H.** genotype effect F _(4, 68)_ = 1.590, *p* = 0.1869, S/E effect F (_1, 68)_ = 25.46, *p* < 0.0001, genotype X S/E interaction F _(4, 68)_ = 0.8135, *p* = 0.5209; **I.** genotype effect F _(4, 68)_ = 1.398, *p* = 0.2441, SP/EP effect F _(1, 68)_ = 34, *p* < 0.0001, genotype X SP/EP interaction F _(4, 68)_ = 0.4236, *p* = 0.7911). No significant difference of duration was found between S vs. E (*p* = 0.5325) or SP vs. EP (*p* = 0.2529) in *Sh3*^−/−^ mice. All other groups of mice showed significantly increased duration in S vs. E or SP vs. EP. * *p* < 0.05, ** *p* < 0.005, *** *p* < 0.0005, **** *p* < 0.0001

To explore whether inflammation plays a role in regression of ASD-associated behaviors, we challenged *Sh3*^+/−^ mice by intraperitoneal injection of LPS (*Sh3*^+/−^+ LPS). Control mice were given the same volume of normal saline (PBS) (*Sh3*^+/−^ + PBS). Both LPS-treated *Sh3*^+/−^ and wild-type (WT) mice experienced weight loss at 24 hours compared to PBS-treated mice (Fig. 1A). This indicated systemic inflammation induced by LPS, consistent with previous reports ^31^. LPS-treated mice regained body weight at 72 hours post-injection, suggesting recovery from the LPS challenge (Fig. 1A). Acute behavioral changes, including decreased locomotor activity ^32^, social avoidance ^33^, and depressive-like behavior ^34^, have been reported in LPS-treated mice around 24 hours post-injection. However, interpretating these behavioral changes in the context of ASD model may be confounded by immediate sickness of test mice ^28,35,36^. Therefore, we performed behavioral analysis two weeks after LPS injection to distinguish “LPS and SHANK3 haploinsufficiency interaction effect” vs. “acute LPS effect” (Fig. 1B). The *Shank3*^Δe4–22^ homozygous (*Sh3*^−/−^) group was included for a comparison ^30^.

To assess the motor coordination, latency to fall from the rotarod was measured in both steady speed (16 rpm) and accelerated speed (4–40 rpm) conditions. As expected, *Sh3*^−/−^ mice showed significantly reduced latency to fall compared to WT+PBS group. No significant difference was observed between WT+PBS and WT+LPS groups, but *Sh3*^+/−^ mice treated with LPS showed significantly shorter latency to fall than the PBS group (Fig. 1C–D). The duration of mouse spent in center of open field test (OFT) showed no significant difference between WT+PBS and WT+LPS groups, but *Sh3*^+/−^+LPS spent significantly less time in the center compared to *Sh3*^+/−^+PBS (Fig. 1E). In the light/dark box test, distance of mouse moved in light chamber showed no significant difference between WT+PBS and WT+LPS groups while *Sh3*^+/−^+LPS significantly moved less in light chamber compared to *Sh3*^+/−^+PBS (Fig. 1F). Repetitive behavior was assayed through scoring grooming behavior. *Sh3*^+/−^+LPS showed significantly longer grooming duration than *Sh3*^+/−^+PBS while WT+PBS and WT+LPS groups were comparable (Fig. 1G). We also conducted a three-chamber social preference test. As expected, *Sh3*^−/−^ mice showed no preference for social targets compared to WT. However, both LPS- and PBS-treated *Sh3*^+/−^ and WT mice spent significantly more time in the chamber with the social target (S) or in the proximity region of the social target (SP) (Fig. 1H–I).

To explore potential sex effect, we compared motor function and sociability in both PBS- and LPS-treated female and male *Sh3*^+/−^ mice. LPS-treated male *Sh3*^+/−^ mice exhibited significant reduced latency to fall in rotarod, although no significant difference found between LPS and PBS group in female *Sh3*^+/−^ mice (Supplementary Fig. 1A–B). Both LPS- and PBS-treated male *Sh3*^+/−^ mice spent significantly more time in S or SP, but no significant difference found in female *Sh3*^+/−^mice (Supplementary Fig. 1C–D). Taken together, our results indicated that, LPS treatment in *Sh3*^+/−^ mice significantly impaired the motor performance on rotarod, changed exploratory or anxiety-like behaviors in OFT and light/dark box test, and increased grooming duration, but did not affect sociability in three-chamber test. By comparison, LPS treatment in WT mice did not have any significant effect on those behaviors. These findings suggest the interplay of inflammation and *Shank3* haploinsufficiency in select domains of behaviors.

**Figure 2.**
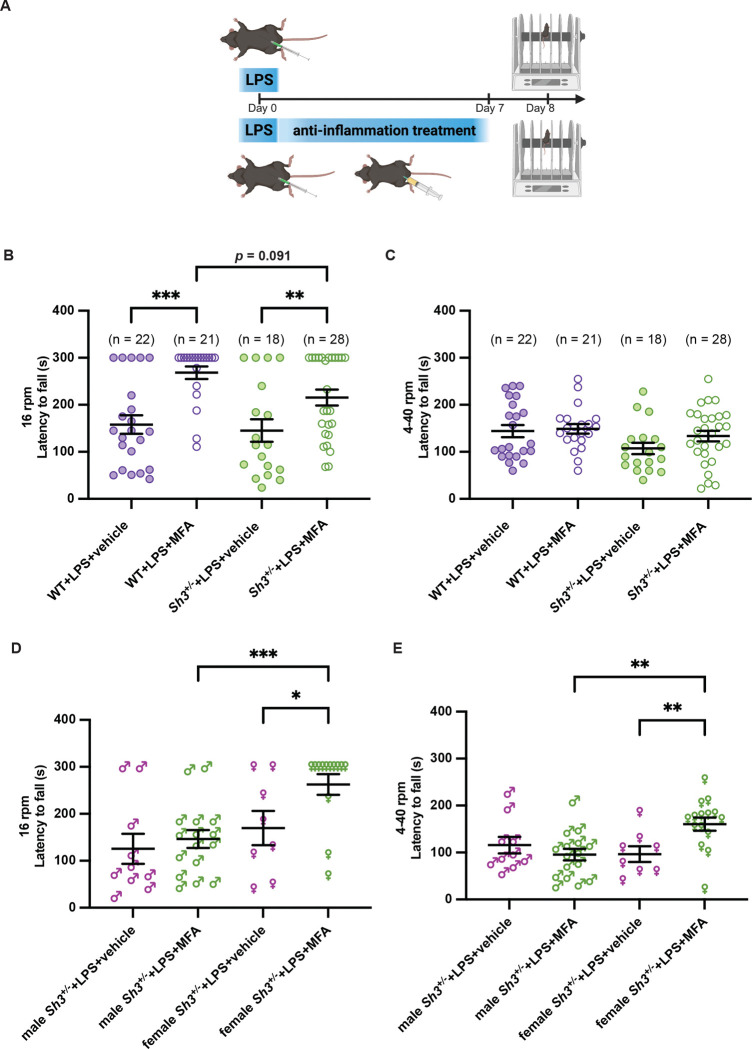
Mefenamic acid (MFA) partially improved the motor behavior deterioration in WT and *Sh3*^+/−^ mice. **A. Schematic drawing for experiment design.** Anti-inflammatory treatment with intraperitoneal MFA injection was given to mice once every day for 7 days after LPS injection. Rotarod tests were performed 24 hours after the last does of MFA injection. **B-C. MFA partially improved the rotarod test performance.** After 1-week treatment of MFA, Steady speed rotarod (16 rpm, **B**) and accelerated speed rotarod (4–40 rpm, **C**) tests were performed and latency to fall was compared. (Two-way ANOVA, **B. 16 rpm,** LPS/MFA effect F _(1, 37)_ = 23.66, *p* < 0.0001, genotype effect F _(1,46)_ = 1.859, *p* = 0.1794, LPS/MFA X genotype interaction F _(1, 37)_ = 0.8697, *p* = 0.3571. **C. 4–40 rpm,** LPS/MFA effect F _(1, 85)_ = 1.721, *p* = 0.1932, genotype effect F _(1,85)_ = 4.822, *p* = 0.0308, LPS/MFA X genotype interaction F _(1, 85)_ = 0.8220, *p* = 0.3672). Compared to vehicle group, MFA-treated *Sh3*^+/−^ and WT mice showed increased latency to fall in 16 rpm rotarod test (*Sh3*^+/−^, *p* = 0.0091; WT, *p* = 0.0002). **D-E. MFA treatment was more effective in female *Sh3***^**+/−**^ **mice than male ones.** Two-way ANOVA was performed to compare sex difference in MFA treatment (**D. 16 rpm,** LPS/MFA effect F _(1, 43)_ = 4.626, *p* = 0.0372, sex effect F _(1,43)_ = 9.227, *p* = 0.004, LPS/MFA X sex interaction F _(1, 43)_ = 1.858, *p* = 0.1799. **E. 4–40 rpm,** LPS/MFA effect F _(1, 16)_ = 2.065, *p* = 0.17, sex effect F _(1,29)_ = 2.369, *p* = 0.1346, LPS/MFA X sex interaction F _(1, 16)_ = 8.046, *p* = 0.0119). MFA-treated female *Sh3*^+/−^ showed significantly higher latency to fall compared to vehicle-treated female *Sh3*^+/−^ (16 rpm, *p* = 0.0229; 40rpm, *p* = 0.0106) and MFA-treated male *Sh3*^+/−^ (16 rpm, *p* = 0.0009; 40rpm, *p* = 0.0011). * *p* < 0.05, ** *p* < 0.005, *** *p* < 0.0005, **** *p* < 0.0001

To assess whether anti-inflammation treatment can reverse the behavioral changes induced by LPS in *Sh3*^+/−^ mice, we applied mefenamic acid (MFA), a nonsteroidal anti-inflammatory drug (NSAID). One of the MFA targets is the intracellular cyclooxygenase (COX) signaling, which can be activated by LPS ^37–39^. Previous study also reported that MFA has neuroprotective effect ^40^. Given that both WT+LPS and *Sh3*^+/^+LPS group exhibited impaired motor function at 1 week after the LPS injection (Supplementary Fig. 2A–B) but such behavior deficits of WT+LPS group recovered to baseline at 2-week post-injection (Fig. 1C–D), we chose day 7 post-injection as the endpoint of MFA treatment (Fig. 2A). We administered MFA via intraperitoneal injection once a day for seven days following LPS injection and then performed rotarod test 24 hours after the final dose of MFA to evaluate the treatment efficacy (Fig. 2A). In the steady speed (16 rpm) rotarod test, both WT+LPS+MFA and *Sh3*^+/−^+LPS+MFA groups showed significantly increased latency to fall compared to vehicle-treated groups (Fig. 2B), suggesting that MFA effectively mitigated the motor function deficits induced by LPS. *Sh3*^+/−^+LPS+MFA showed trend of shorter latency to fall than WT+LPS+MFA (Fig. 2B, *p* = 0.091), suggesting potential less pronounced anti-inflammatory efficacy and the presence of exacerbated inflammatory responses in *Sh3*^+/−^ mice. In contrast, in the accelerated speed (4–40 rpm) rotarod test, MFA did not restore motor function in either WT or *Sh3*^+/−^ mice (Fig. 2C). Moreover, when comparing MFA treatment in female and male *Sh3*^+/−^ mice, MFA significantly increased latency to fall in female *Sh3*^+/−^ mice but not in male *Sh3*^+/−^ mice (Fig. 2D–E). Overall, our results showed that LPS-induced motor function impairment in *Sh3*^+/−^ mice can be partially reversed by anti-inflammatory treatment. Future studies will include additional behavioral assays, as well as time-course and dose-dependent responses to MFA treatment.

### LPS-induced differentially expressed genes (DEGs) were associated with neuroinflammation and neurotransmission in *Sh3*^+/−^ mice.

**Figure 3.**
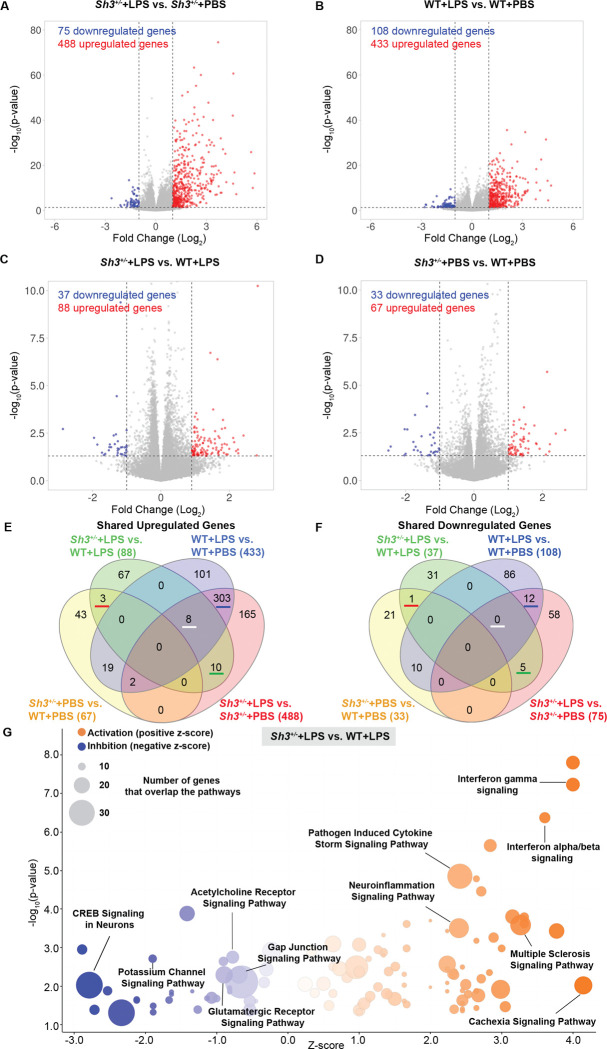
Bulk RNA sequencing revealed distinct molecular mechanism underlying LPS effect on *Sh3*^+/−^ mice. **A-D. The volcano plot shows differentially expressed genes (DEGs) distribution.** DEGs were identified in four comparisons: *Sh3*^+/−^+LPS vs *Sh3*^+/−^+PBS (**A**), WT+LPS vs. WT+PBS (**B**), *Sh3*^+/−^+LPS vs. WT+LPS (**C**), *Sh3*^+/−^+PBS vs. WT+PBS (**D**). Upregulated genes are indicated in red color, downregulated genes are indicated in blue color and DEGs below threshold are indicated in grey color. **E-F. Venn diagrams show shared upregulated and downregulated genes among four comparisons.** Shared upregulated (**E**) and downregulated genes (**F**) numbers are shown within the diagrams. Comparison of *Sh3*^+/−^+LPS vs *Sh3*^+/−^+PBS is red diagram, WT+LPS vs. WT+PBS is blue diagram, *Sh3*^+/−^+LPS vs. WT+LPS is green diagram, and *Sh3*^+/−^+PBS vs. WT+PBS is yellow diagram. **G. Bubble chart shows functions of DEGs of *Sh3***^**+/−**^**+LPS vs. WT+LPS comparison.** Volcano plot of the negative log of p-value (Y-axis) vs. the z-score (X-axis). *p*-value was calculated using the right-tailed Fisher’s Exact Test. A negative log of p-value cutoff of 1.3 (*p* < 0.05) was used to identify significantly changed functional pathways. A z-score range cutoff of ±0.1 was used to identify the activation or inhibition state of a canonical pathway. Some names of pathways are annotated in the chart. Activated pathways are orange bubbles and inhibited pathways are blue bubbles. The size of the bubbles reflects the number of genes overlapped with the pathway database.

To understand how LPS-induced inflammation modifies the penetrance of motor impairment in *Sh3*^+/−^ mice, we analyzed the transcriptomic profiles in the forebrains by bulk RNA-sequencing (RNA-seq) (Supplementary Fig. 3A–B, Supplementary Tables 1 and 2). LPS treatment induced hundreds of differentially expressed genes (DEGs) in both WT and *Sh3*^+/−^ mice compared to PBS-treated groups. The number of upregulated genes (488 DEGs in *Sh3*^+/−^+LPS vs. *Sh3*^+/−^+PBS and 433 DEGs in WT+LPS vs. WT+PBS) was greater than that of downregulated genes (75 DEGs in *Sh3*^+/−^+LPS vs. *Sh3*^+/−^+PBS and 108 DEGs in WT+LPS vs. WT+PBS) (Fig. 3A–B). We identified 88 upregulated and 37 downregulated DEGs between *Sh3*^+/−^+LPS vs. WT+LPS (Fig. 3C). Although we found 67 upregulated and 33 downregulated genes when comparing transcriptomic profiles between *Sh3*^+/−^+PBS and WT+PBS (Fig. 3D), those genes barely overlapped with any LPS-induced DEGs observed in *Sh3*^+/−^ mice. Next, we compared the overlapping genes among four comparisons (Fig. 3E–F, Supplementary Tables 3 and 4). A total of 313 shared upregulated and 12 shared downregulated DEGs were identified between WT+LPS vs. WT+PBS and *Sh3*^+/−^+LPS vs. *Sh3*^+/−^+PBS. Those shared DEGs probably represent the LPS-induced neuroinflammatory responses in mouse brains. Eight upregulated genes shared among *Sh3*^+/−^+LPS vs. WT+LPS, *Sh3*^+/−^+LPS vs. *Sh3*^+/−^+PBS and WT+LPS vs. WT+PBS were well-known for their roles in neuroinflammation (Fig. 3E highlighted with white underline, Table 1). Interestingly, 10 upregulated genes and 5 downregulated genes were found only in *Sh3*^+/−^ mice not in WT mice in response to LPS (Fig. 3E–F highlighted with green underline, Table 2–3), suggesting that LPS-induced neuroinflammation in *Sh3*^+/−^ mice is mediated by distinct signaling pathways. Additionally, when we compared the overlapping genes between *Sh3*^+/−^+PBS vs. WT+PBS and *Sh3*^+/^+LPS vs. WT+LPS, only 4 genes in total (two are protein-coding genes) were identified (Fig. 3E–F highlighted with red underline). Particularly, *Acr* is a *Shank3* subordinate adjacent gene, whose expression increased when *Shank3* is deleted ^41^. To further characterize the function of DEGs from RNA-seq, we performed the pathway analysis using the QIAGEN Ingenuity Pathway Analysis (IPA) ^42^. We identified more activated inflammation-associated pathways in response to LPS challenges in both *Sh3*^+/−^ and WT (Supplementary Fig. 4 and 5). In contrast, inhibited neurotransmission-related pathways including NMDA receptor and calcium signaling pathways were only observed in LPS-treated *Sh3*^+/−^ but not in LPS-treated WT (Supplementary Fig. 4 and 5). To understand the distinct molecular pathways in LPS-treated *Sh3*^+/−^ mice, we delved into the pathway analysis from *Sh3*^+/−^+LPS vs. WT+LPS (Supplementary Fig. 6). The top five activated pathways were all related to inflammatory processes. The top two inhibited pathways, cardiac conduction and CREB signaling in neurons, represent inhibited neuron signal transmission and neuronal functions. The other three inhibited pathways, HIF1α signaling, eNOS signaling and phagosome formation pathways, represent increased neuronal vulnerability to stress and inflammatory stimuli. In addition to the top five pathways with highest z score value, we noticed that most of the upregulated signaling pathways in response to LPS were all inflammation-related, including cytokine storm pathway and neuroinflammation pathway (Fig. 3G). In contrast, the downregulated pathways in LPS-treated *Sh3*^+/−^ mice were neuronal and synaptic function-related, including potassium channel pathway, glutamatergic signaling pathway, acetylcholine signaling pathway, and gap junction signaling pathway (Fig. 3G). Overall, our RNA-seq results suggested that a combination of exacerbated neuroinflammatory responses and impaired neuronal functions is attributed to the LPS-induced behavioral regression in *Sh3*^+/−^ mice.

### Neuroinflammation was increased in *Sh3*^+/−^ mice

**Figure 4.**
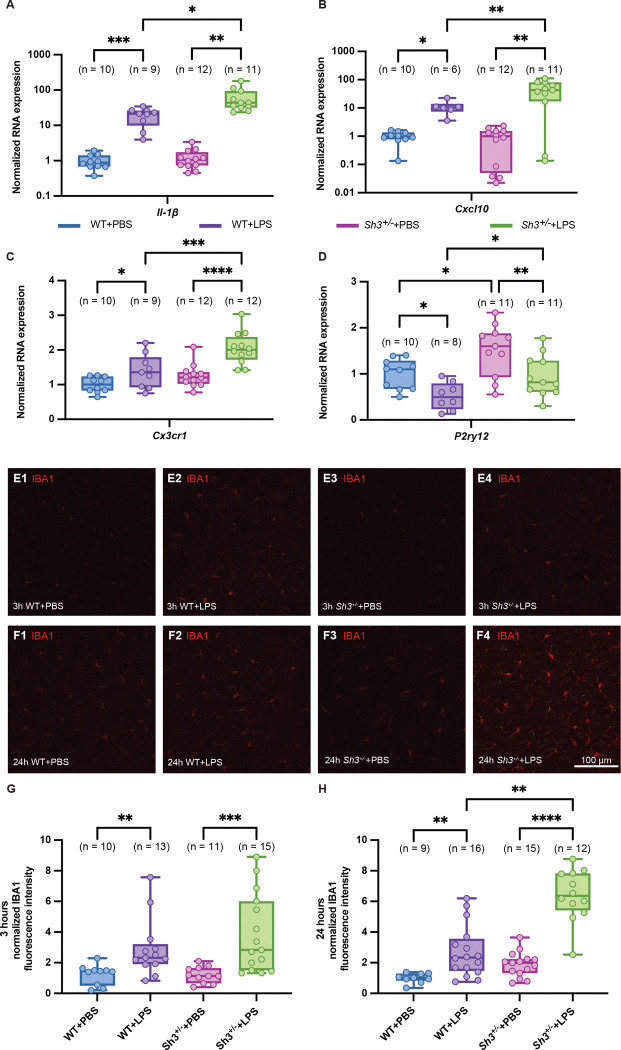
Neuroinflammation increased in *Sh3*^+/−^ mice compared to WT mice in response to LPS. A-D. Differential expression of neuroinflammatory marker genes *Il1b*, *Cxcl10, Cx3cr1* and *P2ry12*. At 24 hours after LPS injection, forebrain RNA was extracted, and RT-qPCR was performed. Target gene expression was normalized to the expression of the housekeeping gene GAPDH. One-way ANOVA was performed to compare gene expression in four groups (*Il1b*: F= 14.93, *p* = 0.0004, *Cxcl10*: F= 16.73, *p* = 0.0002, *Cx3cr1*: F= 14.8, *p* < 0.0001, *P2ry12*: F= 8.442, *p* = 0.0002). As expected, compared to PBS treatment, LPS significantly increased expression of *Il1b, Cxcl10*, and *Cx3cr1*, and decreased expression of *P2ry12* in both WT and *Sh3*^+/−^ mice (WT+PBS vs. WT+LPS, *Il1b*: *p* = 0.0005, *Cxcl10*: *p* = 0.0105, *Cx3cr1*: *p* = 0.0388, *P2ry12*: *p* = 0.0241; *Sh3*^+/−^ +PBS vs. *Sh3*^+/−^ +LPS, *Il1b*: *p* = 0.002, *Cxcl10*: *p* = 0.0015, *Cx3cr1*: *p* < 0.0001, *P2ry12*: *p* = 0.0046). Significant increase of *Il1b, Cxcl10*, *Cx3cr1* was observed in *Sh3*^+/−^+LPS vs. WT+LPS (*Il1b*: *p* = 0.0166, *Cxcl10*: *p* = 0.0076, *Cx3cr1*: *p* = 0.0004). In contrast, significantly increased expression of *P2ry12* was observed in *Sh3*^+/−^+PBS vs. WT+PBS (*p* = 0.0102) and *Sh3*^+/−^+LPS vs. WT+LPS (*p* = 0.036). **E-H. Increased immunohistochemistry fluorescence intensity of IBA1 in *Sh3***^**+/−**^ **+LPS mice.** Mouse brain slices were collected at 3 hours and 24 hours after injection, respectively. Microglia activation was labeled with IBA1 antibody. Quantitative fluorescence intensity of IBA1 at cortex regions from WT+LPS, *Sh3*^+/−^+PBS, and *Sh3*^+/^+LPS were normalized to WT+PBS group and then compared (Kruskal-Wallis test, **G.** *p* = 0.0002; **H.** *p* < 0.0001). LPS increased microglia activation in both WT and *Sh3*^+/−^ mice at both 3 hours and 24 hours after injection compared to PBS groups (WT+PBS vs. WT+LPS, 3 hours, *p* = 0.0067, 24 hours, *p* = 0.0053; *Sh3*^+/−^ +PBS vs. *Sh3*^+/−^ +LPS, 3 hours, *p* = 0.0004, 24 hours, *p* < 0.0001). Moreover, LPS induced significant overactivation of microglia in *Sh3*^+/−^ mice at 24 hours after injection compared to WT mice, not 3 hours (WT+LPS vs. *Sh3*^+/−^ +LPS, 3 hours, *p* = 0.6204, 24 hours, *p* = 0.0017). * *p* < 0.05, ** *p* < 0.005, *** *p* < 0.0005, **** *p* < 0.0001.

To investigate how LPS leads to aggravated neuroinflammation in *Sh3*^+/−^ brains, we first measured the RNA expression of the pro-inflammatory cytokines *Il1b* and chemokines *Cxcl10* using RT-qPCR. *Il1b* and *Cxcl10* have been identified as common upregulated genes across all LPS-treated groups in RNA-seq (Table 1). RT-qPCR confirmed that LPS significantly increased *Il1b* and *Cxcl10* expression in both WT and *Sh3*^+/−^ mice compared to PBS groups. Although no significant difference in *Il1b* and *Cxcl10* expression was found between *Sh3*^+/−^+PBS vs. WT+PBS, *Sh3*^+/−^+LPS showed significantly increased expression compared to WT+LPS group (Fig. 4A–B). A previous study showed that LPS treatment upregulates microglia marker gene *Cx3cr1* while downregulates microglia homeostasis gene *P2ry12* expression ^43^. Therefore, we examined the expression of microglia markers *Cx3cr1* and *P2ry12* using RT-qPCR. We found that, as expected, LPS significantly increased *Cx3cr1* expression and decreased *P2ry12* expression in both WT and *Sh3*^+/−^ mice compared to PBS groups (Fig. 4C–D). However, *Cx3cr1* expression was comparable between *Sh3*^+/−^+PBS and WT+PBS but significantly increased in *Sh3*^+/−^+LPS compared to WT+LPS group (Fig. 4C). In contrast, *P2ry12* expression was significantly higher in *Sh3*^+/−^+PBS than in WT+PBS and higher in *Sh3*^+/−^+LPS than in WT+LPS (Fig. 4D).

We further examined microglia activation by examining the expression of IBA-1, a marker widely used for the assessment of microglia activation state ^44^ (Fig. 4E–H). Compared to PBS treatment, LPS increased IBA-1 staining significantly at 3 hours and 24 hours after LPS treatment in both WT and *Sh3*^+/−^ mice. However, *Sh3*^+/−^+LPS showed significantly higher microglia activation than WT+LPS group at 24 hours but not at 3 hours post-injection. These results indicated that the prolonged activation of microglia contributed to increase of neuroinflammatory responses in *Sh3*^+/−^ mice.

**Figure 5.**
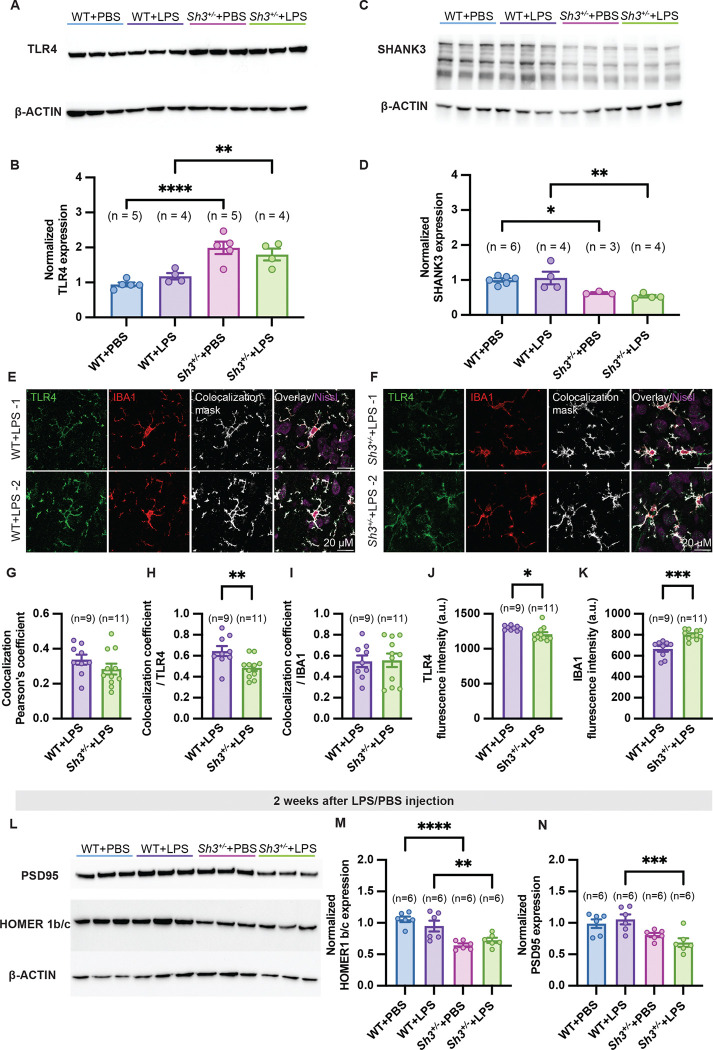
Elevated TLR4 expression and reduced HOMER 1b/c and PSD95 expression in *Sh3*^+/−^ mice. **A-D. Increased TLR4 and decreased SHANK3 protein expression in S*h3*^+/−^ mice.** LPS receptor TLR4 (**A-B**) and SHANK3 (**C-D**) expression were examined in whole-cell lysates using western blot at 24 hours after injection. Target protein expression was normalized to β-ACTIN expression. Quantitative protein expression from WT+LPS, *Sh3*^+/−^+PBS, and *Sh3*^+/−^+LPS were normalized to WT+PBS group and then compared. Significantly elevated TLR4 expression was observed in *Sh3*^+/−^ mice compared to WT mice (WT+PBS vs. *Sh3*^+/−^ +PBS, *p* < 0.0001; WT+LPS vs. *Sh3*^+/−^ +LPS, *p* = 0.0081). As expected, significantly reduced SHANK3 expression was detected in *Sh3*^+/−^ mice (WT+PBS vs. *Sh3*^+/−^ +PBS, *p* = 0.0172; WT+LPS vs. *Sh3*^+/−^ +LPS, *p* = 0.002). **E-J. Colocalization analysis of TLR4/IBA1 between WT+LPS vs. *Sh3***^**+/−**^ **+LPS.** No difference in colocalization Pearson’s coefficient between WT+LPS vs. *Sh3*^+/−^ +LPS was observed (*p* = 0.2309). *Sh3*^+/−^ showed smaller normalized colocalization by TLR4 (*p* = 0.0093) but similar normalized colocalization by IBA1 (*p* = 0.9141) compared to WT. *Sh3*^+/−^ showed decreased TLR4 cellular fluorescence intensity (*p* = 0.0183) but increased IBA1 cellular fluorescence intensity (*p* = 0.0001) than WT. **L-N. Reduced postsynaptic density (PSD) protein HOMER 1b/c and PSD95 expression in *Sh3***^**+/−**^ **mice.** HOMER 1b/c and PSD95 expression were examined in PSD fraction of forebrain using western blot at 2 weeks after injection. Target protein expression was normalized to β-ACTIN expression. Quantitative protein expression from WT+LPS, *Sh3*^+/−^+PBS, and *Sh3*^+/−^+LPS were normalized to WT+PBS group and then compared. Significantly reduced HOMER 1b/c expression was observed in *Sh3*^+/−^ mice (WT+PBS vs. *Sh3*^+/−^ +PBS, *p* < 0.0001; WT+LPS vs. *Sh3*^+/−^+LPS, *p* = 0.0088). Significant decreased PSD95 expression was detected in *Sh3*^+/−^+LPS compared to WT+LPS group (*p* = 0.0007). * *p* < 0.05, ** *p* < 0.005, *** *p* < 0.0005, **** *p* < 0.0001.

Next, to understand the mechanism underlying the LPS-induced microglia overactivation, we analyzed the LPS transduction signaling pathways. LPS is known to bind to Toll-like receptor-4 (TLR-4) ^45^, so we examined TLR4 expression using western blot. We found that TLR4 expression was significantly higher in *Sh3*^+/−^ than WT with either PBS or LPS treatment (Fig. 5A–B). Additionally, we measured the SHANK3 protein expression and found that either PBS- or LPS-treated *Sh3*^+/−^ groups showed a reduction in SHANK3 protein expression compared to WT groups as expected, but no changes in SHANK3 expression were observed after LPS treatment (Fig. 5C–D).

TLR4 is known to associate with LPS-induced microglia activation^46–49^. To investigate whether observed TLR4 augmentation directly facilitates microglia overactivation in LPS treated *Sh3*^+/−^ mice, we performed co-immunostaining of TLR4 and microglia marker IBA1 and analyzed colocalization between TLR4 and IBA1 (Fig. 5E–K). We found that TLR4 and IBA1 expression were well-colocalized in both LPS-treated WT and *Sh3*^+/−^ mice (Fig. 5E–G). However, colocalization coefficient normalized by TLR4 was lower in *Sh3*^+/−^ mice than that of WT mice. The colocalization coefficient normalized by IBA1 was similar between *Sh3*^+/−^ and WT mice (Fig. 5H–I), indicating elevated TLR4 expression in *Sh3*^+/−^ might involve cell types in addition to microglia. Cellular immunofluorescence intensity of IBA1 was significantly higher in *Sh3*^+/−^ IBA1 than that of WT (two-tailed *p* = 0.0001) (Fig. 5K), similar to prior results (Fig. 4H). In contrast, the immunofluorescence intensity of TLR4 in *Sh3*^+/−^ mice is slightly lower than WT (two-tailed *p* = 0.0183) (Fig. 5J) and that is different from the results of immunoblot analysis using fresh mouse brain tissue (Fig. 5A–B). Given that TLR4 was expressed in many different cell types including astrocytes, neutrophils, and brain endothelial cells ^50,51^, these findings supported that augmented TLR4 might also be in other cell types in addition to microglia in *Sh3*^+/−^ brains.

Finally, to validate whether behavior impairment is associated with synaptic dysfunction in *Sh3*^+/−^ mice, we analyzed the expression level of postsynaptic density (PSD) protein HOMER 1b/c and PSD95, which play important roles in synaptic functions^52–54^, using forebrain PSD fraction at two-week post-injection of LPS (Fig. 5L–N). Consistent with our prior report ^30^, HOMER 1b/c was significantly lower in *Sh3*^+/−^ than WT but no significant difference was found when comparing *Sh3*^+/−^+LPS with *Sh3*^+/−^+PBS group (Fig. 5M). In contrast, PSD95 was comparable between WT+PBS and *Sh3*^+/−^+PBS groups but significantly reduced in *Sh3*^+/−^+LPS group compared to WT+LPS group. (Fig. 5N). These results indicate that LPS treatment in SHANK3 haploinsufficiency mice causes a significant but selective reduction of PSD95 protein that is known to be important for synaptic functions and may contribute to the worsen behaviors in LPS treated Sh3^+/−^ mice.

## Discussion

**Figure 6.**
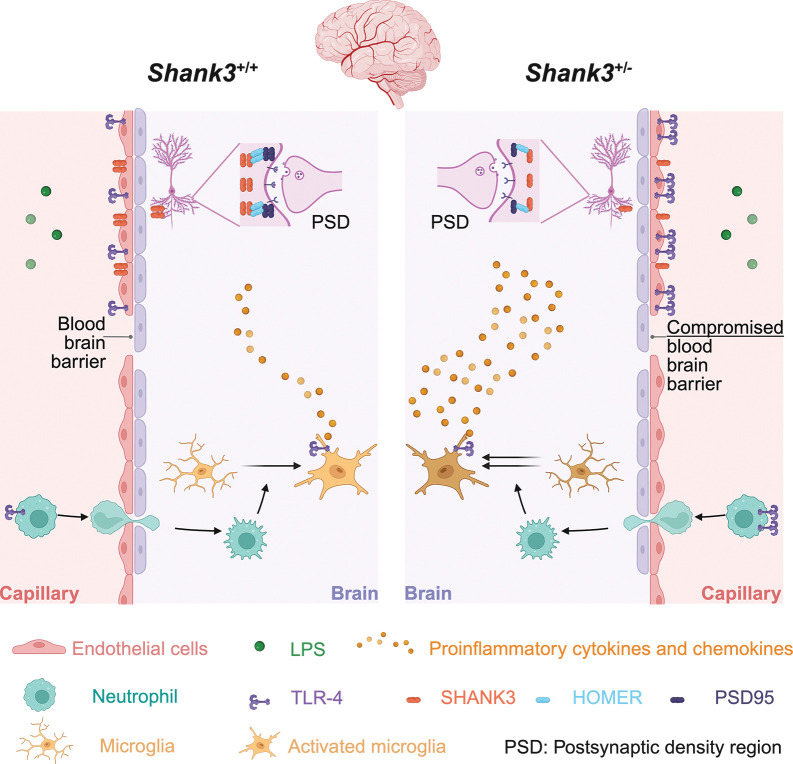
Summary of increased neuroinflammation susceptibility in *Sh3*^+/−^ mice.

In this study, we successfully established a preclinical model and experimental protocol to assess the role of neuroinflammation induced by LPS in *Sh3*^+/−^ mice (Fig. 6). We demonstrated that LPS challenge modified the penetrance of motor function deficits, anxiety-like behavior, and repetitive behavior but did not alter social preference in *Sh3*^+/−^ mice. More interestingly, the motor and emotion specific behavioral changes are reminiscent of catatonia features that are often observed in humans with *SHANK3* deficiency and idiopathic ASD ^71–73^. Additionally, our extensive comparative transcriptome analysis among the different groups validated the experimental protocol of LPS induction and revealed distinct underlying molecular mechanisms. The significantly upregulated genes ubiquitously associated with inflammatory pathways in LPS treated groups indicated the effectiveness of LPS induction. The downregulated genes in multiple pathways associated with neuronal and synaptic functions strongly support a potential causal link between LPS treatment and increased penetrance of abnormal behaviors in *Sh3*^+/−^ mice. Moreover, the changes of the expression of microglia markers and increased activation of microglia after LPS treatment are consistent with the role of neuroinflammation in the brain. Finally, the partial reversal of motor impairment in LPS treated *Sh3*^+/−^ mice through the anti-inflammation treatment with MFA further supports this causality. Notably, MFA treatment did not restore LPS-treated WT and *Sh3*^+/−^ mice performance in the accelerating speed rotarod. These findings suggest that the mechanisms underlying inflammation-induced motor impairment are different between steady speed and accelerating speed rotarod tests ^74^. Additionally, one-week MFA treatment was more effective in female *Sh3*^+/−^ mice than male mice (Fig. 2D–E) and LPS-treated male *Sh3*^+/−^ mice performance was worse than female ones (Supplementary Fig. 1A–B), indicating potential sex difference in motor function^75^ and neuroinflammation process^76^.

The behavior impairments induced by LPS in *Sh3*^+/−^ mice could attribute to the dysfunction of neuronal cells resulted from neuroinflammation. In the previous study that our lab contributed, LPS challenge increased the excitability in D1 but not D2 medium spiny neurons using whole-cell patch clamp recordings and decreased neuron calcium transients in Nucleus Accumbens in response to social stimuli using *in vivo* Ca^2+^ imaging^28^. Our data showed that increased neuroinflammation downregulated genes associated with neuronal and synaptic functions in *Sh3*^+/−^ mice compared to WT mice and reduced synaptic protein PSD95 in *Sh3*^+/−^ mice.

LPS-induced neuroinflammation could also alter the function of non-neuronal cells in *Sh3*^+/−^ mice. Our findings of augmented TLR4 expression and microglia activation associated with LPS treatment suggested that microglia are primarily involved in more significant neuroinflammatory responses in *Sh3*^+/−^ mice. Consistent with the findings in many reports in literature supporting that TLR4 mediates microglial activation in brains ^46–49^, our findings also support that the upregulation of TLR4 contributes to the observed microglial activation following LPS administration. The direct causality between upregulation of TLR4 and microglia activation due to LPS induction has been demonstrated in a recent study^77^. Our observations are consistent with this finding and support the augmented TLR4 as the cause of microglia activation in SHANK3 deficiency mice. Further analysis of TLR4 and IBA1 cellular colocalization indicated that additional cell types might also be involved in LPS-TLR4 (ligand-receptor) mediated neuroinflammation. Our previous study showed that SHANK3 deficiency in periphery nerve system contributes to the exaggerated inflammatory responses^78^. Our recently published collaborative study has shown that conditional knock-out of SHANK3 major isoform in brain endothelial cells recapitulated social behavior impairment and other synaptic phenotypes ^79^. Our findings are aligned with previous studies showing that *Shank3* deficiency exacerbates the inflammatory responses in peripheral neurons either induced by LPS ^29,78^ or by maternal immune activation during the pregnancy ^80^. Overall, our findings showed that LPS exacerbated neuroinflammation in *Sh3*^+/−^ mouse brains through unique molecular mechanisms as summarized in Fig. 6 for further investigations in future.

In humans, haploinsufficiency of *SHANK3* is highly penetrant for neurodevelopmental impairments ^81^. Approximately ~70% of PMS and *SHANK3* deficiency patients met the diagnostic criteria for ASD. The comorbidity of intellectual disability, anxiety, and epilepsy are highly penetrant or common ^72^. There is an increasing recognition of behavioral regression and catatonia among young teens with *SHANK3* deficiency ^71^. However, the triggers and mechanisms underlying the behavioral regression are largely unknown. Although few clinical case reports implicated neuroinflammation as a trigger ^21,82^, our findings may offer a mechanistic explanation for the behavioral regressions observed in *SHANK3* patients after experiencing a subclinical infection responded to the immune modulator treatment. Our study may support a link between neuroinflammation and autism susceptibility in general.

Our results establish an important proof-of-concept study to dissect the link between neuroinflammation and autism susceptibility. The apparent limitations of our studies include that we have not provided definitive causality for the cell types and specific molecular mechanism directly implicated in the increased neuroinflammatory responses indued by LPS in SHANK3 haploinsufficiency mice. Future studies are warranted to examine dosage- or treatment duration-dependent effects of LPS on behavioral outcomes at different neurodevelopmental stages and in different sexes of *Shank3* deficiency mice. Despite these limitations, our results suggest that *Shank3* haploinsufficiency increases susceptibility to neuroinflammation-induced behavior impairment through distinct molecular pathways. These findings provide new insights into the role of neuroinflammation in ASD and for developing anti-inflammatory treatments as both preventive and therapeutic strategies for behavior regression in ASD that are warranted for further investigations.

## Materials and methods

### Animals

Animals were housed under standard 12 h/12 h light/dark cycles (7 AM-7 PM) with food, water ad libitum, controlled temperature and humidity in the Yale University animal facility. Experiments procedures conducted were following the Institutional Animal Care and Use Committee (IACUC) protocol at Yale University (protocol No. 2025–20271). *Shank3*^Δe4–22^ mouse line (Jax Strain #:039524) was generated in-house and had been crossed with C57BL/6J mouse line (Jax Strain #:000664) over 20 generations. Wild-type (*WT*), *Shank3*^Δe4–22^ homozygous (*Sh3*^−/−^) and *Shank3*^Δe4–22^ heterozygous mice (*Sh3*^+/−^) were obtained through heterozygotes breeding as previously described ^30^. All experiment procedures were performed within a time frame of 9 AM-5 PM. Animals of the same sex were group housed (2–5 mice per cage). Both male and female mice were used. One cage of mice with same genotype and same sex were assigned to PBS or LPS group randomly. Experimental order of different group was randomized. Mice from multiple groups for same experiments were carried out at same time. Before experiments, each mouse was cataloged with identification number in an excel sheet which included detailed information such as genotype and drug treatment. During experiments, experimenter handled mice based on its identification number and attempted to be semi-blinded. Post-processing, data handling and statistical analysis were semi-automated performed using software stated below. Mice with health problems such as runt, fight wounds, malocclusion, and eye problems, were excluded from experiments. For RNA and protein extraction, mice were deeply anesthetized with 5% isoflurane, then mouse brains were quickly collected, flash frozen in liquid nitrogen and stored in −80 °C freezer until further use. The number of animals used in each experiment was determined based on our lab prior experience and a prior power analysis was calculated using G*power statistics software^83^ (Supplementary table 5). Total number of mice used for each experiment was as follows: 86 mice for MFA treatment rotarod test, 52–95 mice for open field, rotarod and three-chamber behavior test, 49–66 mice for light/dark box and grooming behavior test, 15 mice for RNA sequencing, 40 mice for quantitative PCR, 24 mice for wide-field IBA1 staining, 6 mice for TLR4/IBA1 co-staining, 18 mice for whole-cell protein analysis and 24 mice for synaptic protein analysis. All experiments were replicated at least once, except that RNA sequencing was done one time.

### Drug

Three to five-week-old female and male mice were intraperitoneally injected with LPS (Sigma, L6511) in saline at 1 mg/kg or same volume of saline (PBS). To test the mefenamic acid (MFA) anti-inflammatory effect on behavior improvement, MFA (Thermofisher, AAJ6270522) dissolved in DMSO at 5 mg/kg or the same volume of DMSO (vehicle) was injected intraperitoneally to the mice with LPS injection once a day for 7 days. Before injection, freshly made stock solution of MFA in DMSO (MFA treatment) or DMSO (vehicle treatment) was diluted with 1:100 ratio in PBS to minimize DMSO toxicity.

### Behavior assay

For behavioral experiments, mice were handled for 3 days and habituated in the procedure room for 1 hour before behavioral assays. All behavioral tests were conducted once for each mouse.

Anxiety-like behaviors were tested by open field test (OFT) and light/dark box test followed previous studies^30,84^. In OFT, mouse spontaneous activity in Noldus Open Field (4 chambers, 40 (L) × 40 (W) × 30 (H) cm per chamber) over 10 minutes was recorded with EthoVision XT video tracking software (Noldus). The Noldus two-chambered light/dark box is equipped with the lid of dark chamber made by infrared translucent material. The dimensions of the compartment are one third for the dark chamber (20 (L) × 20 (W) × 20 (H) cm) and two thirds for the light chamber (20 (L) × 40 (W) × 20 (H) cm). Mice were firstly placed into the open-light chamber (~700 lux) and given 5 min to freely explore both light and dark (0 lux) chambers. The video was recorded with EthoVision XT video tracking software. Ethovision software was used to automatically score the travel distance and duration in arena.

Repetitive behavior was tested by evaluating mouse grooming behaviors. Mouse was placed in a Noldus phenotyper (30 (L) × 30 (W) × 45 (H) cm) and recorded with EthoVision XT video tracking software. Mouse freely explored for 10 minutes and last 5 minutes of grooming behavior was hand scored.

Motor coordination was tested by rotarod (Med Associate) performance as described ^30,85^. Mice were tested in two 5 min trials with an inter-trial interval of 30 min. In accelerating session, revolutions per minute (rpm) of the turning barrel is accelerating from 4 to 40. In steady speed session, the rpm of the turning barrel is fixed at 16. Latency to fall, or to rotate off the top of the turning barrel, was measured by the timer.

Social preference was tested by a three-chamber assay as described^85^. An unfamiliar age- and sex-matched mouse was used as a social stimuli mouse in the three-chamber test and habituated under a wired enclosure for 3 days before testing. A customized chamber composed of a rectangular Plexiglas arena (42 (L) × 56.5(W) × 35.8 (H) cm) divided into three chambers (each chamber 18.5 × 42 cm), with 2 doors connecting chambers and two enclosures (wired pencil cups with metal vertical bars) located in the two outer chambers. Briefly, the test mouse was habituated in the center chamber for 5 min. Then the social stimuli mouse was placed under one of the pencil cups and 2 doors were lifted allowing the experimental mouse to freely explore the whole arena. The location of the empty and mouse enclosures, and the social stimuli mouse were changed and counterbalanced for each trial. The EthoVision XT video tracking software was used to score the duration of mouse spending in the empty chamber, the mouse chamber, proximity regions (5 cm in distance) close to the empty enclosure, and proximity regions close to the mouse enclosure.

### Bulk RNA sequencing

Total RNA was isolated from the forebrain hemisphere using Trizol (Invitrogen, 15596018) followed manufacturer protocol and then treated with DNase I (Invitrogen, AM1907) to remove contaminating DNA. RNA samples were sent to Yale Center for Genome Analysis (YCGA) for bulk RNA sequencing (RNA-seq). See details about sequencing process in Supplementary Information. After obtaining the data, low quality reads were trimmed, and adaptor contamination were removed using Trim Galore (v0.5.0). Trimmed reads were mapped to the mouse reference genome (mm10) using HISAT2 (v2.1.0) ^86^. Gene expression levels were quantified using StringTie (v1.3.3b) ^87^. Differentially expressed genes were identified using DESeq2 (v 1.22.1) ^88^. Unpaired *t*-test *p* value was used for filtering DEGs with significant differences between the two groups. Log_2_fold cutoff threshold at 1 and *p* < 0.05 was used to filter DEGs from two groups comparison (WT+LPS vs. WT+PBS, *Sh3*^+/−^+LPS vs. *Sh3*^+/−^+PBS, *Sh3*^+/^+LPS vs. WT+LPS and *Sh3*^+/−^+PBS vs. WT+PBS) for genes comparison and analysis. *Pathway analysis*: QIAGEN Ingenuity Pathway Analysis (IPA) library of canonical pathways was used to identify the pathways most significant to the data set. Molecules from the dataset meeting the criteria in which log_2_fold cutoff threshold at 0.25 and *p* < 0.05 were associated with a canonical pathway in the QIAGEN Knowledge Base were considered for the analysis. Because using log_2_fold cutoff threshold at 1 only revealed three activated pathways in *Sh3*^+/^+LPS vs. WT+LPS comparison which were all inflammation-related, so we expanded the log_2_fold cutoff threshold to 0.25. The significance of the association between the data set and the canonical pathway was measured based on: 1) a ratio of the number of molecules from the data set that map to the pathway divided by the total number of molecules that map to the canonical pathway was displayed, 2) a right-tailed Fisher’s Exact Test was used to calculate a p-value determining the probability that the association between the genes in the dataset and the canonical pathway was explained by chance alone, 3) A z-score was calculated to indicate the likelihood of activation or inhibition of that pathway. In this paper, pathways with right-tailed Fisher’s Exact Test *p* < 0.05, z-score > 0.1 was considered as activated pathways and z-score < −0.1 was considered as inhibited pathways. The volcano plot was created using VolcaNoseR ^89^. Veen diagram was plotted using InteractiVenn ^90^.

### Real-Time Quantitative Reverse Transcription PCR (RT-qPCR)

Total RNA was isolated using same protocol as RNA-seq. RNA was transcribed into cDNA using iScript cDNA Synthesis Kit (Bio-Rad, 1708890) and qPCR was performed in triplicate using iQ SYBR Green Supermix (Bio-Rad, 1708880). Gene expressions were normalized to the expression of GAPDH. Primers used to amplify cDNAs are: *Il1b,* forward, TGGACCTTCCAGGATGAGGACA, reverse, GTTCATCTCGGAGCCTGTAGTG; *Cxcl10,* forward, ATCATCCCTGCGAGCCTATCCT, reverse, GACCTTTTTTGGCTAAACGCTTTC; *Cx3cr1,* forward, GAGCATCACTGACATCTACCTCC, reverse, AGAAGGCAGTCGTGAGCTTGCA; *P2ry12* forward, CATTGACCGCTACCTGAAGACC, reverse, GCCTCCTGTTGGTGAGAATCATG; *Gapdh* forward, CAAAATGGTGAAGGTCGGTG, reverse, AATGAAGGGGTCGTTGATGG.

### Immunohistochemistry

Transcardiac perfusion was conducted at both 3 hours and 24 hours after LPS or PBS injection with 20 mL PBS then 20 mL 4% paraformaldehyde (PFA, Santa Cruz, sc-281692). Mouse brains were collected, fixed in 4% PFA for 24 hours, then changed to 30% (w/v) sucrose before sectioning. 50 μM brain sections between Bregma 1 mm to −1 mm were obtained using vibratome (Leica, VT1200S). Primary antibody anti-Rabbit-IBA1 (1:200, FUJIFILM Wako Chemicals, 019–19741), anti-Mouse-TLR4 (1:100, Abcam, ab22048), and secondary antibody Donkey Anti-Mouse Alexa Fluor^®^ 488 (1:500, Abcam, ab150105), Donkey anti-Rabbit Alexa Fluor^™^ 594 (1:500, Invitrogen, A-21207) were used. Zeiss LSM 980 confocal microscopy with 20X objective len was used for wide-field IBA1 imaging. ImageJ was used for IBA1 fluorescence intensity quantification. Total 49 images at 3 hours and 52 images at 24 hours were analyzed (Fig. 4E–H). Zeiss LSM 900 confocal microscopy with 63X objective len was used for TLR4/IBA1 co-staining imaging and ZEN Blue software was used for colocalization and fluorescence intensity analysis. Total 20 images were analyzed (Fig. 5E–J). Colocalization analysis was performed on a pixel-by-pixel basis. Every pixel in the image was plotted in the scatter diagram based on its intensity level from each channel. With setting the threshold in each channel, a scatter plot with four quadrants was generated. Same threshold was used across all images and pixels in colocalized quadrant were used for generating white “Colocalization mask” images and further analysis. Software automatically measured colocalization coefficient (Pearson’s coefficient) and fluorescence intensity in each channel. Colocalization coefficient normalized by TLR4 or IBA1 was calculated as sum of intensity of all pixels in colocalized quadrant divided by sum of intensity of all pixels above the threshold value in TLR4 channel or IBA1 channel ($\frac{\sum {\mathrm{SumGreyCh}}_{1\mathrm{colocalized}}}{\sum {\mathrm{SumGreyCh}}_{1\mathrm{total}}}$). Fluorescence intensity was calculated as sum of all gray values from channel divided by the total number of pixels in this channel ($\frac{\sum {\mathrm{GreyCh}1}_{i}}{\mathrm{AreaCh}1}$). Immunostaining and imaging were done under same condition and same setting at same time across different groups. Biological triplicates of brain slices from one mouse brain were employed. Results from at least three mice brains per group were analyzed and compared.

### Western blot

Western blot was performed as previously described ^91^. Whole cell lysates from mouse forebrain hemisphere were prepared using 1x lysis buffer (Cell Signaling Technology, 9803S) containing 1x protease/phosphatase inhibitor (Cell Signaling Technology, 5872S). Crude postsynaptic density (PSD) fraction from mouse forebrain hemisphere were prepared following previously described protocol with some modifications^30,92^. See details in Supplementary Information. Samples were boiled in 4x Laemlli buffer (Bio-Rad, 161–0747) at 98 °C for 5 min before gel electrophoresis. Home-made anti-rabbit SHANK3 C-terminus monoclonal antibody (1:1000), anti-rabbit TLR4 monoclonal antibody (1:1000, Santa Cruz, sc-293072), anti-mouse PSD95 monoclonal antibody (1:1000, Invitrogen, MA1–046), HOMER 1b/c monoclonal HRP-conjugated antibody (1:1000, Santa Cruz, sc-17842-HRP), and β-ACTIN monoclonal HRP-conjugated antibody (1:1000, Invitrogen, MA5–15739-HRP) were used.

### Statistical analysis

Graphpad Prism 10.0 (GraphPad Software) was used for the statistical analysis and data plot. Differences were analyzed by student’s *t*-test if comparing two variables or using ANOVA if comparing more than two variables followed by multiple comparisons either using statistical hypothesis testing with correction or planned comparisons without correction. Significance was determined by two-tailed *t*-test *p* value or multiplicity adjusted *p* value. For data which was not sampled from Gaussian distribution, a nonparametric test was performed (Fig. 4G–H). Since unequal variance observed in LPS-evoked cytokines and chemokines, Brown-Forsythe and Welch ANOVA tests were performed (Fig. 4A–B). See detailed statistical analysis for each figure in Supplementary Table 5. n represents the total number of data points per group, usually meaning the mice number with an exception that n represents brain slice numbers (Fig. 4G–H and Fig. 5G–K). Both sexes of mice were used and balanced across genotype and treatment, with an exception that WT+PBS and WT+LPS groups were all male mice in RNA-seq experiments. Behavioral difference between male and female mice were explored in Supplementary Fig. 1. In Fig. 1 and Fig. 4, individual data points were plotted as box plot: whiskers go down to the smallest value and up to the largest value, box extends from the 25th to 75th percentiles and line in the middle of the box is plotted at the median. In other figures, individual data points were plotted and presented as average ± SEM. *p < 0.05* was considered statistically significant. BioRender was used to create schematic diagram.

## Supplementary Material

Supplement 1

Supplement 2

Supplement 3

Supplement 4

Supplement 5

Supplement 6

## Figures and Tables

**Table 1.** Eight Upregulated DEGs shared among *Sh3*^+/−^+LPS vs. WT+LPS, *Sh3*^+/−^+LPS vs. *Sh3*^+/−^+PBS and WT+LPS vs. WT+PBS.

Gene name	Protein function
*Bcl3*	BCL3 is an autonomous determinant of memory/terminal effector cell balance during CD8^+^ T cell differentiation in response to acute viral infection%^55^.
*Ccl3*	CCL3 as a cytokine contributes to progressive tissue damage and functional impairment during secondary injury after spinal cord injury %^56^.
*Cxcl10*	CXCL10 as a lymphocyte chemoattractant contributes to the intraparenchymal trafficking of lymphocytes during acute CNS inflammation %^57^.
*Gbp5*	GBP5 as a GTPase participates in the regulation of immunity and neuroinflammation %^58^.
*Ifitm6*	IFITM6 encodes cell surface proteins that modulate cell-cell adhesion and cell differentiation, is induced in various macrophages, and increases expression 24 hours after LPS %^59^.
*Il1b*	IL-1β can be acutely induced in the brain following peripheral bacterial infections, and microglia facilitate exaggerated IL-1β production upon exposure to acute systemic inflammation induced by bacterial LPS %^60^.
*Steap4*	STEAP4 as a key effector molecule contributes to the pathogenesis of neuroinflammation in experimental autoimmune encephalomyelitis %^61^.
*Tlr2*	Toll-like receptors (TLRs) are fundamental sensor molecules of the host innate immune system, which detect conserved molecular signatures of a wide range of microbial pathogens and initiate innate immune responses via distinct signaling pathways %^62^.

**Table 2.** Ten upregulated DEGs only shared between *Sh3*^+/−^+LPS vs. WT+LPS and *Sh3*^+/−^+LPS vs. *Sh3*^+/−^+PBS, not include in WT+LPS vs. WT+PBS.

Gene name	Protein function
*Alms1-ps1*	ALMS1, centrosome and basal body associated, pseudogene 1.
*Atf3*	ATF3 as a transcription factor responds to and exert dual effects on inflammatory responses and in involved in neuroinflammatory response %^63^.
*Ccl12*	CCL12 as a chemokine expressed by microglia is involved in inflammation %^64^.
*Fas*	Fas/FasL pathway plays a key role in immune homeostasis and leads to excessive neuroinflammation during excessive neuroinflammation %^65^.
*Gm11681*	long non-coding RNA.
*Hspa1a*	HSPA1A and HSPA1B are two subtypes of HSPA1 proteins. HSPA1 is a member of the stress-inducible 70-kDa heat shock protein (HSP70) family, which plays a critical role in the cellular stress response ^66,67^.
*Hspa1b*	
*Hspb1*	
*Oas1g*	OAS1G is an important murine antiviral response factor, function through activating RNase L to degrade viral RNA in turn inhibiting viral replication and propagation %^68^.
*Prok2*	PROK2 is a secreted protein involved in the pathogenesis of several acute and chronic neurological diseases. It shows that PROK2 mediates neuronal cell deaths in traumatic brain injury via ferroptosis %^69^.

**Table 3.** Five downregulated DEGs only shared between *Sh3*^+/−^+LPS vs. WT+LPS and *Sh3*^+/−^+LPS vs. *Sh3*^+/−^+PBS, not include in WT+LPS vs. WT+PBS.

Gene name	Protein function
*4930415F15Rik*	SPMIP7 protein is mainly involved in spermatogenesis but is also expressed in the brain and its function is unclear.
*AC173482.5*	Predicted gene, Gm47797
*Gm14393*	Predicted gene
*Gm5111*	Predicted gene
*Tgif2*	TGIF2 is a transcriptional repressor. Downregulation of TGIF2 is correlated with neuronal apoptosis and development of autism in mice %^70^.

## References

[1] Regier D. A., Kuhl E. A. & Kupfer D. J. The DSM-5: Classification and criteria changes. World Psychiatry 12, 92–98 (2013). 10.1002/wps.20050 23737408 PMC3683251

[2] Stefanatos G. A. Regression in autistic spectrum disorders. Neuropsychol Rev 18, 305–319 (2008). 10.1007/s11065-008-9073-y 18956241

[3] Manelis L. Language regression is associated with faster early motor development in children with autism spectrum disorder. Autism Res 13, 145–156 (2020). 10.1002/aur.2197 31468744

[4] Boterberg S., Van Coster R. & Roeyers H. Characteristics, Early Development and Outcome of Parent-Reported Regression in Autism Spectrum Disorder. J Autism Dev Disord 49, 4603–4625 (2019). 10.1007/s10803-019-04183-x 31463633

[5] Kohlenberg T. M. Psychiatric illness and regression in individuals with Phelan-McDermid syndrome. J Neurodev Disord 12, 7 (2020). 10.1186/s11689-020-9309-6 32050889 PMC7014655

[6] Ghaziuddin M. Catatonia: A Common Cause of Late Regression in Autism. Front Psychiatry 12, 674009 (2021). 10.3389/fpsyt.2021.674009 34777033 PMC8585308

[7] Betancur C. & Buxbaum J. D. SHANK3 haploinsufficiency: a “common” but underdiagnosed highly penetrant monogenic cause of autism spectrum disorders. Mol Autism 4, 17 (2013). 10.1186/2040-2392-4-17 23758743 PMC3695795

[8] Satterstrom F. K. Large-Scale Exome Sequencing Study Implicates Both Developmental and Functional Changes in the Neurobiology of Autism. Cell 180, 568–584 e523 (2020). 10.1016/j.cell.2019.12.036 31981491 PMC7250485

[9] Zhou X. Integrating de novo and inherited variants in 42,607 autism cases identifies mutations in new moderate-risk genes. Nat Genet (2022). 10.1038/s41588-022-01148-2 PMC9470534 35982159

[10] Fu J. M. Rare coding variation provides insight into the genetic architecture and phenotypic context of autism. Nat Genet (2022). 10.1038/s41588-022-01104-0 PMC9653013 35982160

[11] Nevado J. Variability in Phelan-McDermid Syndrome in a Cohort of 210 Individuals. Front Genet 13, 652454 (2022). 10.3389/fgene.2022.652454 35495150 PMC9044489

[12] Soorya L. Prospective investigation of autism and genotype-phenotype correlations in 22q13 deletion syndrome and SHANK3 deficiency. Mol Autism 4, 18 (2013). 10.1186/2040-2392-4-18 23758760 PMC3707861

[13] Leblond C. S. Meta-analysis of SHANK Mutations in Autism Spectrum Disorders: a gradient of severity in cognitive impairments. PLoS Genet 10, e1004580 (2014). 10.1371/journal.pgen.1004580 25188300 PMC4154644

[14] Serrada-Tejeda S., Martínez-Piédrola R. M., Máximo-Bocanegra N., Sánchez-Herrera-Baeza P. & Pérez-de-Heredia-Torres M. Descriptive Analysis of Adaptive Behavior in Phelan-McDermid Syndrome and Autism Spectrum Disorder. Frontiers in neuroscience 16, 893003 (2022). 10.3389/fnins.2022.893003 35864987 PMC9295709

[15] Reierson G. Characterizing regression in Phelan McDermid Syndrome (22q13 deletion syndrome). J Psychiatr Res 91, 139–144 (2017). 10.1016/j.jpsychires.2017.03.010 28346892 PMC5469716

[16] Denayer A. Neuropsychopathology in 7 Patients with the 22q13 Deletion Syndrome: Presence of Bipolar Disorder and Progressive Loss of Skills. Molecular Syndromology 3, 14–20 (2012). 10.1159/000339119 22855650 PMC3398818

[17] Zwanenburg R. J., Ruiter S. A. J., van den Heuvel E. R., Flapper B. C. T. & Van Ravenswaaij-Arts C. M. A. Developmental phenotype in Phelan-McDermid (22q13.3 deletion) syndrome: a systematic and prospective study in 34 children. Journal of Neurodevelopmental Disorders 8, 16 (2016). 10.1186/s11689-016-9150-0 27118998 PMC4845478

[18] Serret S. Lithium as a rescue therapy for regression and catatonia features in two SHANK3 patients with autism spectrum disorder: case reports. BMC Psychiatry 15, 107 (2015). 10.1186/s12888-015-0490-1 25947967 PMC4428105

[19] Phelan K. & McDermid H. E. The 22q13.3 Deletion Syndrome (Phelan-McDermid Syndrome). Mol Syndromol 2, 186–201 (2012). 10.1159/000334260 22670140 PMC3366702

[20] Phelan K. Phelan-McDermid syndrome: a classification system after 30 years of experience. Orphanet J Rare Dis 17, 27 (2022). 10.1186/s13023-022-02180-5 35093143 PMC8800328

[21] Bey A. L. Subacute Neuropsychiatric Syndrome in Girls With SHANK3 Mutations Responds to Immunomodulation. Pediatrics 145, e20191490 (2020). 10.1542/peds.2019-1490 32015180 PMC7802010

[22] Durand C. M. Mutations in the gene encoding the synaptic scaffolding protein SHANK3 are associated with autism spectrum disorders. Nat Genet 39, 25–27 (2007). 10.1038/ng1933 17173049 PMC2082049

[23] He G. Dexmedetomidine attenuates neuroinflammation and microglia activation in LPS-stimulated BV2 microglia cells through targeting circ-Shank3/miR-140–3p/TLR4 axis. Eur J Histochem 67 (2023). 10.4081/ejh.2023.3766 PMC10476535 37491974

[24] Fischer I. Hyperbaric Oxygen Therapy Alleviates Social Behavior Dysfunction and Neuroinflammation in a Mouse Model for Autism Spectrum Disorders. Int J Mol Sci 23 (2022). 10.3390/ijms231911077 PMC9570008 36232395

[25] Cope E. C. Immature Neurons and Radial Glia, But Not Astrocytes or Microglia, Are Altered in Adult Cntnap2 and Shank3 Mice, Models of Autism. eNeuro 3 (2016). 10.1523/eneuro.0196-16.2016 PMC5066262 27785461

[26] Urrutia-Ruiz C. Deletion of the Autism-Associated Protein SHANK3 Abolishes Structural Synaptic Plasticity after Brain Trauma. Int J Mol Sci 23 (2022). 10.3390/ijms23116081 PMC9181590 35682760

[27] Uchino S. & Waga C. Novel Therapeutic Approach for Autism Spectrum Disorder: Focus on SHANK3. Curr Neuropharmacol 13, 786–792 (2015). 10.2174/1570159×13666151029105547 26511836 PMC4759317

[28] Tzanoulinou S. Inhibition of Trpv4 rescues circuit and social deficits unmasked by acute inflammatory response in a Shank3 mouse model of Autism. Molecular Psychiatry 27, 2080–2094 (2022). 10.1038/s41380-021-01427-0 35022531 PMC9126815

[29] Zhang L. SHANK3 in vagal sensory neurons regulates body temperature, systemic inflammation, and sepsis. Front Immunol 14, 1124356 (2023). 10.3389/fimmu.2023.1124356 36845137 PMC9944123

[30] Wang X. Altered mGluR5-Homer scaffolds and corticostriatal connectivity in a Shank3 complete knockout model of autism. Nat Commun 7, 11459 (2016). 10.1038/ncomms11459 27161151 PMC4866051

[31] Piirsalu M. Treatment With Lipopolysaccharide Induces Distinct Changes in Metabolite Profile and Body Weight in 129Sv and Bl6 Mouse Strains. Frontiers in Pharmacology 11 (2020). 10.3389/fphar.2020.00371 PMC7118216 32292347

[32] Jung H. LPS induces microglial activation and GABAergic synaptic deficits in the hippocampus accompanied by prolonged cognitive impairment. Scientific Reports 13, 6547 (2023). 10.1038/s41598-023-32798-9 37085584 PMC10121592

[33] Decker Ramirez E. B. The effects of lipopolysaccharide exposure on social interaction, cytokine expression, and alcohol consumption in male and female mice. Physiology & Behavior 265, 114159 (2023). 10.1016/j.physbeh.2023.114159 36931488 PMC10121933

[34] O’Connor J. C. Lipopolysaccharide-induced depressive-like behavior is mediated by indoleamine 2,3-dioxygenase activation in mice. Mol Psychiatry 14, 511–522 (2009). 10.1038/sj.mp.4002148 18195714 PMC2683474

[35] Parrott J. M., Oster T. & Lee H. Y. Altered inflammatory response in FMRP-deficient microglia. iScience 24, 103293 (2021). 10.1016/j.isci.2021.103293 34820601 PMC8602000

[36] Hodges S. L. Lipopolysaccharide-induced inflammation leads to acute elevations in pro-inflammatory cytokine expression in a mouse model of Fragile X syndrome. Physiol Behav 215, 112776 (2020). 10.1016/j.physbeh.2019.112776 31838149 PMC12990187

[37] Eliopoulos A. G., Dumitru C. D., Wang C. C., Cho J. & Tsichlis P. N. Induction of COX-2 by LPS in macrophages is regulated by Tpl2-dependent CREB activation signals. Embo j 21, 4831–4840 (2002). 10.1093/emboj/cdf478 12234923 PMC126277

[38] Blais V., Turrin N. P. & Rivest S. Cyclooxygenase 2 (COX-2) inhibition increases the inflammatory response in the brain during systemic immune stimuli. J Neurochem 95, 1563–1574 (2005). 10.1111/j.1471-4159.2005.03480.x 16277613

[39] Cimolai N. The potential and promise of mefenamic acid. Expert Review of Clinical Pharmacology 6, 289–305 (2013). 10.1586/ecp.13.15 23656341

[40] Khansari P. S. & Halliwell R. F. Evidence for neuroprotection by the fenamate NSAID, mefenamic acid. Neurochemistry International 55, 683–688 (2009). 10.1016/j.neuint.2009.06.014 19563851

[41] Lu X. Transcriptional determinism and stochasticity contribute to the complexity of autism-associated SHANK family genes. Cell Rep 43, 114376 (2024). 10.1016/j.celrep.2024.114376 38900637 PMC11328446

[42] Krämer A., Green J., Pollard J. Jr. & Tugendreich S. Causal analysis approaches in Ingenuity Pathway Analysis. Bioinformatics 30, 523–530 (2014). 10.1093/bioinformatics/btt703 24336805 PMC3928520

[43] Sousa C. Single-cell transcriptomics reveals distinct inflammation-induced microglia signatures. EMBO Rep 19 (2018). 10.15252/embr.201846171 PMC6216255 30206190

[44] Norden D. M., Trojanowski P. J., Villanueva E., Navarro E. & Godbout J. P. Sequential activation of microglia and astrocyte cytokine expression precedes increased Iba-1 or GFAP immunoreactivity following systemic immune challenge. Glia 64, 300–316 (2016). 10.1002/glia.22930 26470014 PMC4707977

[45] Tiwari P. C. & Pal R. The potential role of neuroinflammation and transcription factors in Parkinson disease. Dialogues Clin Neurosci 19, 71–80 (2017). 10.31887/DCNS.2017.19.1/rpal 28566949 PMC5442366

[46] Cartarozzi L. P., de Melo Lima B. H., Tomiyama A. L. R. M., Santos F. R. & de Oliveira A. L. R. Toll-like receptors 2 and 4 influence neuronal survival and glial reactions following ventral root crush injury in mice. Journal of Neuroimmunology 406, 578655 (2025). 10.1016/j.jneuroim.2025.578655 40505343

[47] Yao L. Toll-like receptor 4 mediates microglial activation and production of inflammatory mediators in neonatal rat brain following hypoxia: role of TLR4 in hypoxic microglia. Journal of Neuroinflammation 10, 785 (2013). 10.1186/1742-2094-10-23 PMC3575244 23388509

[48] Jiang Y. Evobrutinib mitigates neuroinflammation after ischemic stroke by targeting M1 microglial polarization via the TLR4/Myd88/NF-κB pathway. Mol Med 31, 148 (2025). 10.1186/s10020-025-01203-8 40263985 PMC12016189

[49] Zhu J. Toll-like receptor 4 deficiency in Purkinje neurons drives cerebellar ataxia by impairing the BK channel-mediated after-hyperpolarization and cytosolic calcium homeostasis. Cell Death Dis 15, 594 (2024). 10.1038/s41419-024-06988-w 39147737 PMC11327311

[50] TLR4 expression in the Human Protein Atlas, <https://www.proteinatlas.org/ENSG00000136869-TLR4/single+cell> (

[51] Sjöstedt E. An atlas of the protein-coding genes in the human, pig, and mouse brain. Science 367, eaay5947 (2020). 10.1126/science.aay5947 32139519

[52] Levy A. M., Gomez-Puertas P. & Tümer Z. Neurodevelopmental Disorders Associated with PSD-95 and Its Interaction Partners. Int J Mol Sci 23 (2022). 10.3390/ijms23084390 PMC9025546 35457207

[53] Jung S. & Park M. Shank postsynaptic scaffolding proteins in autism spectrum disorder: Mouse models and their dysfunctions in behaviors, synapses, and molecules. Pharmacol Res 182, 106340 (2022). 10.1016/j.phrs.2022.106340 35792298

[54] Shiraishi-Yamaguchi Y. & Furuichi T. The Homer family proteins. Genome Biology 8, 206 (2007). 10.1186/gb-2007-8-2-206 17316461 PMC1852408

[55] Jaiswal H. The NF-κB regulator Bcl-3 restricts terminal differentiation and promotes memory cell formation of CD8+ T cells during viral infection. PLoS Pathog 17, e1009249 (2021). 10.1371/journal.ppat.1009249 33508001 PMC7872245

[56] Pelisch N., Rosas Almanza J., Stehlik K. E., Aperi B. V. & Kroner A. CCL3 contributes to secondary damage after spinal cord injury. J Neuroinflammation 17, 362 (2020). 10.1186/s12974-020-02037-3 33246483 PMC7694914

[57] Klein R. S. Regulation of neuroinflammation: the role of CXCL10 in lymphocyte infiltration during autoimmune encephalomyelitis. J Cell Biochem 92, 213–222 (2004). 10.1002/jcb.20052 15108349

[58] Zhong K. A Synthetic Steroid 5α-Androst-3β, 5, 6β-triol Alleviates Radiation-Induced Brain Injury in Mice via Inhibiting GBP5/NF-κB/NLRP3 Signal Axis. Mol Neurobiol 61, 4074–4089 (2024). 10.1007/s12035-023-03831-9 38057643

[59] Han J. H. IFITM6 expression is increased in macrophages of tumor-bearing mice. Oncol Rep 25, 531–536 (2011). 10.3892/or.2010.1092 21165565

[60] Cunningham C., Wilcockson D. C., Campion S., Lunnon K. & Perry V. H. Central and systemic endotoxin challenges exacerbate the local inflammatory response and increase neuronal death during chronic neurodegeneration. J Neurosci 25, 9275–9284 (2005). 10.1523/jneurosci.2614-05.2005 16207887 PMC6725757

[61] Zhao J. STEAP4 expression in CNS resident cells promotes Th17 cell-induced autoimmune encephalomyelitis. J Neuroinflammation 18, 98 (2021). 10.1186/s12974-021-02146-7 33879167 PMC8059164

[62] Lester S. N. & Li K. Toll-like receptors in antiviral innate immunity. J Mol Biol 426, 1246–1264 (2014). 10.1016/j.jmb.2013.11.024 24316048 PMC3943763

[63] Li Y. The multifaceted roles of activating transcription factor 3 (ATF3) in inflammatory responses - Potential target to regulate neuroinflammation in acute brain injury. J Cereb Blood Flow Metab 43, 8–17 (2023). 10.1177/0271678x231171999 37165649 PMC10638996

[64] Ross J. L. Microglia and monocyte-derived macrophages drive progression of pediatric high-grade gliomas and are transcriptionally shaped by histone mutations. Immunity 57, 2669–2687.e2666 (2024). 10.1016/j.immuni.2024.09.007 39395421 PMC11578068

[65] Krzyzowska M., Kowalczyk A., Skulska K., Thörn K. & Eriksson K. Fas/FasL Contributes to HSV-1 Brain Infection and Neuroinflammation. Front Immunol 12, 714821 (2021). 10.3389/fimmu.2021.714821 34526992 PMC8437342

[66] Wu S. HSPA1A Protects Cells from Thermal Stress by Impeding ESCRT-0–Mediated Autophagic Flux in Epidermal Thermoresistance. Journal of Investigative Dermatology 141, 48–58.e43 (2021). 10.1016/j.jid.2020.05.105 32533962

[67] Kampinga H. H. Guidelines for the nomenclature of the human heat shock proteins. Cell Stress and Chaperones 14, 105–111 (2009). 10.1007/s12192-008-0068-7 18663603 PMC2673902

[68] Frankiw L., Mann M., Li G., Joglekar A. & Baltimore D. Alternative splicing coupled with transcript degradation modulates OAS1g antiviral activity. Rna 26, 126–136 (2020). 10.1261/rna.073825.119 31740586 PMC6961538

[69] Bao Z. Prokineticin-2 prevents neuronal cell deaths in a model of traumatic brain injury. Nat Commun 12, 4220 (2021). 10.1038/s41467-021-24469-y 34244497 PMC8270965

[70] Lei J., Deng Y. & Ma S. Downregulation of TGIF2 is possibly correlated with neuronal apoptosis and autism-like symptoms in mice. Brain Behav 12, e2610 (2022). 10.1002/brb3.2610 35592894 PMC9226810

[71] Dhossche D. Early-onset catatonia associated with SHANK3 mutations: looking at the autism spectrum through the prism of psychomotor phenomena. Front Psychiatry 14, 1186555 (2023). 10.3389/fpsyt.2023.1186555 37810596 PMC10557257

[72] Srivastava S. Updated consensus guidelines on the management of Phelan-McDermid syndrome. Am J Med Genet A 191, 2015–2044 (2023). 10.1002/ajmg.a.63312 37392087 PMC10524678

[73] Yurumez E., Temelturk R. D., Kaymak B., Karabıyıkoğlu Y. & Zengin N. D. Catatonia in autism spectrum disorders: Prevalence, functional impact, and diagnostic insights. Psychiatry Res 342, 116250 (2024). 10.1016/j.psychres.2024.116250 39488943

[74] Monville C., Torres E. M. & Dunnett S. B. Comparison of incremental and accelerating protocols of the rotarod test for the assessment of motor deficits in the 6-OHDA model. J Neurosci Methods 158, 219–223 (2006). 10.1016/j.jneumeth.2006.06.001 16837051

[75] Hernandez A. R., Truckenbrod L. M., Campos K. T., Williams S. A. & Burke S. N. Sex differences in age-related impairments vary across cognitive and physical assessments in rats. Behav Neurosci 134, 69–81 (2020). 10.1037/bne0000352 31886694 PMC7078049

[76] Han J., Fan Y., Zhou K., Blomgren K. & Harris R. A. Uncovering sex differences of rodent microglia. Journal of Neuroinflammation 18, 74 (2021). 10.1186/s12974-021-02124-z 33731174 PMC7972194

[77] Hines D. J., Choi H. B., Hines R. M., Phillips A. G. & MacVicar B. A. Prevention of LPS-induced microglia activation, cytokine production and sickness behavior with TLR4 receptor interfering peptides. PLoS One 8, e60388 (2013). 10.1371/journal.pone.0060388 23555964 PMC3610686

[78] Han Q. SHANK3 Deficiency Impairs Heat Hyperalgesia and TRPV1 Signaling in Primary Sensory Neurons. Neuron 92, 1279–1293 (2016). 10.1016/j.neuron.2016.11.007 27916453 PMC5182147

[79] Kim Y. E. Endothelial SHANK3 regulates tight junctions in the neonatal mouse blood-brain barrier through β-Catenin signaling. Nat Commun 16, 1407 (2025). 10.1038/s41467-025-56720-1 39915488 PMC11802743

[80] Atanasova E. Immune activation during pregnancy exacerbates ASD-related alterations in Shank3-deficient mice. Mol Autism 14, 1 (2023). 10.1186/s13229-022-00532-3 36604742 PMC9814193

[81] Leblond C. S. Meta-analysis of SHANK Mutations in Autism Spectrum Disorders: A Gradient of Severity in Cognitive Impairments. PLOS Genetics 10, e1004580 (2014). 10.1371/journal.pgen.1004580 25188300 PMC4154644

[82] Jesse S. Phelan McDermid Syndrome: Multiple Sclerosis as a Rare but Treatable Cause for Regression-A Case Report. Int J Mol Sci 22 (2021). 10.3390/ijms22052311 PMC7956287 33669083

[83] Faul F., Erdfelder E., Lang A. G. & Buchner A. G*Power 3: a flexible statistical power analysis program for the social, behavioral, and biomedical sciences. Behav Res Methods 39, 175–191 (2007). 10.3758/bf03193146 17695343

[84] Bourin M. & Hascoët M. The mouse light/dark box test. European Journal of Pharmacology 463, 55–65 (2003). 10.1016/S0014-2999(03)01274-3 12600702

[85] Moy S. S. Mouse behavioral tasks relevant to autism: phenotypes of 10 inbred strains. Behav Brain Res 176, 4–20 (2007). 10.1016/j.bbr.2006.07.030 16971002 PMC1857288

[86] Kim D., Paggi J. M., Park C., Bennett C. & Salzberg S. L. Graph-based genome alignment and genotyping with HISAT2 and HISAT-genotype. Nature Biotechnology 37, 907–915 (2019). 10.1038/s41587-019-0201-4 PMC7605509 31375807

[87] Pertea M. StringTie enables improved reconstruction of a transcriptome from RNA-seq reads. Nature Biotechnology 33, 290–295 (2015). 10.1038/nbt.3122 PMC4643835 25690850

[88] Love M. I., Huber W. & Anders S. Moderated estimation of fold change and dispersion for RNA-seq data with DESeq2. Genome Biol 15, 550 (2014). 10.1186/s13059-014-0550-8 25516281 PMC4302049

[89] Goedhart J. & Luijsterburg M. S. VolcaNoseR is a web app for creating, exploring, labeling and sharing volcano plots. Scientific Reports 10, 20560 (2020). 10.1038/s41598-020-76603-3 33239692 PMC7689420

[90] Heberle H., Meirelles G. V., da Silva F. R., Telles G. P. & Minghim R. InteractiVenn: a web-based tool for the analysis of sets through Venn diagrams. BMC Bioinformatics 16, 169 (2015). 10.1186/s12859-015-0611-3 25994840 PMC4455604

[91] Wang S. E. TRPV1 Regulates Stress Responses through HDAC2. Cell Rep 19, 401–412 (2017). 10.1016/j.celrep.2017.03.050 28402861

[92] Carlin R. K., Grab D. J., Cohen R. S. & Siekevitz P. Isolation and characterization of postsynaptic densities from various brain regions: enrichment of different types of postsynaptic densities. J Cell Biol 86, 831–845 (1980). 10.1083/jcb.86.3.831 7410481 PMC2110694

